# Socio-demographic and economics factors associated with suicide mortality in Iran, 2001–2010: application of a decomposition model

**DOI:** 10.1186/s12939-018-0794-0

**Published:** 2018-06-14

**Authors:** Hassan Haghparast-Bidgoli, Giulia Rinaldi, Hossein Shahnavazi, Hamid Bouraghi, Aliasghar A. Kiadaliri

**Affiliations:** 1 Institute for Global Health, University College London, 30 Guilford Street, London, WC1N 1EH UK; 2 St.George’s University of London, London, UK; 3 Iranian Forensic Medicine Organization, Tehran, Iran; 4 Department of Health Information Technology, School of Allied Medical Sciences, Hamadan University of Medical Sciences, Hamadan, Iran; 5 Department of Clinical Sciences Lund, Orthopaedics, Clinical Epidemiology Unit, Lund University, Faculty of Medicine, Lund, Sweden

**Keywords:** Suicide mortality, Temporal variation, Spatial analysis, Panel data, Iran

## Abstract

**Background:**

Suicide is a major global health problem, especially among youth. Suicide is known to be associated with a variety of social, economic, political and religious factors, vary across geographical and cultural regions. The current study aimed to investigate the effects of socioeconomic factors on suicide mortality rate across different regions in Iran.

**Methods:**

The data on distribution of population and socio-economic factors (such as unemployment rate, divorce rate, urbanization rate, average household expenditure etc.) at province level were obtained from the Statistical Centre of Iran and the National Organization for Civil Registration. The data on the annual number of deaths caused by suicide in each province was extracted from the published reports of the Iranian Forensic Medicine Organization. We used a decomposition model to distinguish between spatial and temporal variation in suicide mortality.

**Results:**

The average rate of suicide mortality was 5.5 per 100,000 population over the study period. Across the provinces (spatial variation), suicide mortality rate was positively associated with household expenditure and the proportion of people aged 15–24 and older than 65 years and was negatively associated with the proportion of literate people. Within the provinces (temporal variation), higher divorce rate was associated with higher suicide mortality. By excluding the outlier provinces, the results showed that in addition to the proportion of people aged 15–24 and older than 65, divorce and unemployment rates were also significant predictors of spatial variation in suicide mortality while divorce rate was associated with higher suicide mortality within provinces.

**Conclusion:**

The findings indicate that both spatial and temporal variations in suicide mortality rates across the provinces and over time are determined by a number of socio-economic factors. The study provides information that can be of importance in developing preventive strategies.

**Electronic supplementary material:**

The online version of this article (10.1186/s12939-018-0794-0) contains supplementary material, which is available to authorized users.

## Background

Suicide is a major global health problem, especially among youth, where suicide is in the top three causes of death in people aged 10–24 years [1]. Overall, suicide and self-harm is the 18th highest cause of disability adjusted life years (DALYs) loss globally [2]. Evidence also shows that DALYs loss contributed to self-harm has decreased by 17% from 2005 to 2015 and that the death rates due to self-harm have also decreased by 16.3% between 1980 and 2015. Although rates of fatal suicide have found to be overall decreasing in countries across the world from the 1990s to the present [3–8], this is not the case in certain countries and age groups [9–13].

Countries with the highest suicide rates are Eastern European neighbors (including Russia, Estonia, Latvia and Lithuania) as well as countries such as Sri Lank, Korea and Cuba [14]. Teenagers and young adults carry the high rates of suicide mortality in these countries with 46.5 and 23.6 suicide deaths per 100,000 population in Sri Lanka and Russia, respectively, recorded per year amongst 15–19 year olds [15]. Suicide rate in the Eastern Mediterranean Region (EMR) countries is reported to be lower than the average global suicide rates, ranging from 0.55 to 5.4 per 100,000 population [16]. However, mental disorders, such as depression and anxiety in the EMR region are present at a higher rate than average global level [9].

In Iran, suicide rates have increased from 4.4 to 5.2 per 100,000 population from 2006 to 2010, with the largest burden being amongst males and amongst those 20 to 29 years old [12, 17]. Fatal suicide attempts were higher in males, but non-fatal attempts were higher in females [18]. Around 83% of deaths by suicide in Iran are in urban areas [18] and among them 79.9% of the individuals were not working (including housewives, students or unemployed men) [19]. Suicide rates in age groups are vague with evidence reporting the highest suicide rates amongst 15–65 year olds [18].

Suicide is known to be affected by a variety of social, economic, political and religious factors in different populations [20–22]. Factors associated with suicide vary across geographical and cultural regions. Contradictory evidence shows that lower socioeconomic status can be positively or negatively associated with suicide rates depending on the location [22–32]. Similarly, certain populations have the highest suicide rates amongst the young [12, 24, 33] and others amongst the elderly [7, 25, 34, 35]. Studies assessing associations of suicide have been carried out in many countries [21, 22, 24–30, 36–38], but there is a need to explore the associations of suicide within Iran. This study aims to investigate the effects of socio-demographic and economic factors on suicide mortality rate across and within different regions in Iran. It specifically, assessed spatial and temporal variations in suicide rate across different provinces, for a period of 10 years (2001–2010).

## Methods

### Data and data sources

The data on the distribution of population by province and year were obtained from the Statistical Centre of Iran [39]. Data on annual suicide mortality in each province were collected from the published reports of the Iranian Forensic Medicine Organization (IFMO) affiliated to the Judicial Authority in Iran [40]. The IFMO is responsible for recording all suicide cases in a national registry. All recorded suicide cases are certified by autopsy [41]. We then calculated the suicide rate per 100,000 people by province and year.

Annual province-specific data on socio-demographic and economic variables including unemployment rate, divorce rate, urbanization rate, average household expenditure, literacy rate, and percentage of male in population were collected from the Statistical Centre of Iran for all 28 provinces for the study period (2001–2010). It should be noted that in 2004, a province (Khorasan province) split in 3 provinces and we pooled data for these three provinces for years 2005–2010.

### Statistical analysis

A decomposition model, similar to Phillips [42] was used for the data analysis. This model was used in order to differentiate between spatial (across provinces) and temporal (over time) variations in suicide mortality rates and to determine how socio-demographic and economic factors associate with suicide mortality rates in these two contexts. The model is expressed as follows:

$$ {y}_{it}=\alpha +\beta {X}_i+\eta\ \left({x}_{it}-{X}_i\right)+{\nu}_i+{\gamma}_t+{\varepsilon}_{it} $$

**y**_**it**_ represents suicide mortality rate; **α** is the intercept; **β** captures the effect of between-province differences, represented by province means (shown in capital letters) for a specific characteristic over the entire period; **x**_**it**_ represent the explanatory variables for each province i and year t; **η** measures the effect of within-province changes, i.e. province-year deviations from the overall province mean. Therefore, the **β** coefficients show how socio-demographic and economic factors are associated with cross-sectional change in suicide mortality rates (across provinces) and the **η** coefficients show how the covariates associate with temporal variation in suicide mortality rates (over time within provinces). The model also includes a province-specific residual term, **ν**_**i**_**,** which is handled as a random variable and allows correlation among observations from the same province, γ_t_ are year-specific dummy variables to capture aggregate time effects that influence all provinces, and a residual error term, **ε**_**it**_, which permits correlation over time among observations from the same province. The model was estimated using maximum likelihood techniques with robust standard errors [42].

## Results

Table 1 presents descriptive statistics for dependent and independents variables. Between 2001 and 2010, the mean suicide mortality rate across the 28 provinces, was 5.47 per 100,000. The variation in suicide mortality rates across provinces was greater than those within states over time, indicated by the larger spatial standard deviation compared to the temporal standard deviation.

**Table 1:** Descriptive statistics for dependent and independent variables

Variables	Mean	SD overall	SD spatial	SD temporal	Min	Max
Suicide per 100,000	5.47	3.76	3.63	1.20	0.99	23.84
Divorce rate	1.12	0.46	0.36	0.30	0.21	2.49
Urbanization, %	63.45	12.07	12.05	2.26	41.50	95.00
Unemployment, %	12.13	3.72	2.62	2.68	4.10	30.68
Annual expenditure per head (10,000 IRR^a^)	2382.43	1200.76	480.79	1103.69	566.01	6353.80
Literacy ^b^	83.11	4.24	4.31	0	68.03	91.27
Male, % ^b^	50.85	0.67	0.68	0	49.73	52.81
Aged 15–24 years, % ^b^	25.77	1.31	1.33	0	22.08	28.16
Aged 65+,% ^a^	5.19	0.95	0.97	0	2.95	7.30

^a^1 USD was around 10,000 Iranian Rial (IRR) in 2010

^b^Time-invariant variables. Data for these variables was available for 2006 only

There are also substantial differences in economic variables across and within the provinces over time. For example, urbanization rate with standard deviation of 12 and 2.3% varies considerably across provinces and over time, respectively.

Table 2 shows the cross-sectional and temporal variations in the suicide mortality rate and the covariates between 2001 and 2010. Detailed summary statistics for all the variables and for each province are presented in Additional file 1. Overall, there are substantial variations in suicide mortality rate and covariates across the provinces in both 2001 and 2010. For example, in 2001, suicide mortality rate ranges from 1 in 100,000 population to 14 and this gap has increase substantially in 2010 (Table 2).

**Table 2:** Cross-sectional and temporal variations in summary statistics for dependent and time-varying independent variables over the study period

Variable	2001	2010	% Change
	Mean (range)	Mean (range)	
Suicide mortality rate per 100,000 population	5.0 (1–13.9)	5.8 (1.9–23.8)	17%
Divorce rate (per 1000 people)	0.8 (0.2–1.5)	1.6 (0.6–2.5)	106%
Unemployment rate	15.9 (7.8–30.7)	13 (8.5–20.5)	−18%
Household equivalent per-capita expenditure (per million IRR^a^)	9.2 (5.7–16.9)	42.8 (21.5–63.5)	364%
Urbanization rate	60.4 (41.5–92.1)	66.2 (49.5–95)	9%

^a^1 USD was around 10,000 Iranian Rial (IRR) in 2010

There are substantial temporal changes in the suicide mortality rate and all the covariates between 2001 and 2010. Suicide mortality rate has increased by 17%, from around 5 per 100,000 population in 2001 to around 6 in 2010. All economic indicators have improved substantially during this time period. For example, household equivalent per-capita expenditure tripled and unemployment rate decreased by 18% by 2010. Moreover, divorce rate was nearly doubled by 2010 (Table 2).

The findings from the decomposition analysis are presented in Table 3. The results show that across the provinces, those with higher per capita household expenditure, lower literacy rate, and those with higher proportion of youth (aged 15–24) and 65+ populations have higher suicide mortality rates.

**Table 3:** Results of decomposition models

Decomposition model	Model 1 ^a^		Model 2 ^a, b^	
	Across provinces	Over time	Across provinces	Over time
Divorce rate (per 1000 people)	1.993	1.586**	4.313***	1.582**
Unemployment rate	0.407	−0.040	0.394**	− 0.017
Household equivalent per capita expenditure (per million IRR^c^)	0.003**	0.000	0.000	0.000
Urbanization rate	0.018	−0.046	− 0.045	− 0.066
Literacy rate ^d^	− 0.358*		− 0.170	
Percentage male ^d^	−0.564		0.426	
Percentage aged 15–24 ^d^	1.495**		0.736	
Percentage aged 65+ ^d^	0.973		1.152**	
Ln(population)	−1.131		−0.177	
Intercept	20.207		−31.522	

***,**,* significant at 1, 5 and 10%, respectively

a. Accounted for time variable and first order autocorrelation

b. Excluding Ilam province from the sample as outlier

c. * 1 USD was around 10,000 Iranian Rial (IRR) in 2010

d. Time-invariant variables. Data for these variables was available for 2006 only

Looking at the effect of social covariates on the temporal variation in suicide mortality rate, results show that divorce rate is positively associated with temporal variation in suicide mortality rate, i.e., within each province, suicide mortality rate increases when divorce rate increased. Economic factors (i.e. per capita household expenditure and unemployment rate) do not affect temporal variation in overall suicide mortality rates.

By excluding Ilam province from the analysis (Model 2), household expenditure and literacy rate no longer predict suicide mortality across provinces. Under this model, the provinces with higher divorce rate and unemployment rate have higher suicide mortality rates.

## Discussion

The findings of the current study demonstrate that both geographic and temporal variation in suicide mortality rate are closely tied to several varying social conditions across provinces and over time. The mean suicide rate of 5.47 per 100,000 is in line with previous Iranian studies which state between 4.2–6.7 per 100,000 people [12, 17, 19, 43]. The suicide rate varied in Iran more greatly amongst different provinces than overtime, with an overall 17% increase in suicide over the study period. Similarly, the divorce rates, household average expenditures and urbanization rates increased overall during the study period.

### Spatial trends

Across all the provinces, increased household expenditure, lower literacy rate, higher proportion of youth (15–24 age group) and elderly population (over 65) were significantly associated with the suicide mortality.

Despite, small effect size, the direct positive association between the household expenditure and suicide mortality was in contradiction with the a number of previous studies conducted in different settings in East Asia and Western Europe [44–46], which reported that a higher household expenditure was associated with lower suicide rates. However, this finding was in line with the studies conducted in Brazil [47] and Italy [48]. In addition, a systematic review of association between suicide and geographical socio-economic characteristics by Rehkopf and Buka [22] demonstrated that 70% of studies included showed an inverse relationship between income and suicide, whilst, 30% showed a positive relationship. In fact, in Model 2 of the current study, where Ilam is removed, higher household expenditure is no longer a significant predictor of geographic variation of suicide mortality. This could be because Ilam has the highest suicide rate in the country and its average household expenditure was not amongst the highest 50%. Therefore, this results might be due to omitted variable bias or other potential factors that are not included in the analysis, such as prevalence of drug abuse, psychiatric disorders etc.

Across the provinces, lower literacy rate is found to be a weak predictor of suicide mortality in Model 1, but not significantly associated to suicide across provinces in Model 2. Previous evidence [49–51] has shown that lower literacy rates predict higher suicide rates, which contradicts the fact that lower socioeconomic status is linked to suicide [52]. However, arguments can be made that this could be due to better data in locations with higher literacy rates or that higher literacy rate could mean a greater understanding of one’s own socioeconomic disadvantage [52].

Having more young people (aged 15–24) or older people over 65 was significantly associated with higher suicide rates in both Model 1 and 2 across all provinces in Iran. Research demonstrates that often populations with large proportion of elderly or young adults has significantly raised suicide rates [8]. Consequently, spatial differences in the demographics of Iran are significantly correlated to suicide rates. This agrees with previous evidence from 2005 in Ilam where 74% of suicides occurred in individuals under the age of 29 and only 3% were in those over 50 years old as well as from another 2012 Iranian study demonstrating 77% of suicide occurred in those under 30 years old [53, 54]. This pattern is often seen more in developing countries, with developed countries having a tendency to see a rise in suicide in the elderly population [55] . The data from this study, being more recent, demonstrates an increase in suicide rates related among the older age group, which may reflect in some extent due to recent economic growth in Iran, parallel to increasing proportion of the elderly population.

### Temporal trends

Divorce rate was the only factor that was positively associated with suicide mortality over time in all the provinces. An Iranian suicidology systematic review study has previously found a strong association amongst family conflict and marital problems with suicide attempts in Iran [56]. Further supporting this results, studies from high income settings also have found strong positive associations between divorce rate and suicide demonstrating the impact of social well-being on suicide [42, 57]. In Iran, this has been harder to demonstrate, as divorce rate is very low compared to western countries and divorcees who attempt suicide are in low numbers [19, 58, 59]. The increasing temporal trend in suicide rate in Iran has shown an association with increasing divorce rate, which, supports known theories.

The temporal trends of increasing urbanization and household expenditure did not significantly predict suicide mortality rates in Iran. Similarly, the decrease in unemployment over the study period did not significantly predict suicide mortality rates. There may be various reasons and confounders to why no direct prediction may be seen between these values and suicide, ranging from changes to mental health care access in urbanized area to reasons related to unemployment benefits. However, according to our results no significant relationship was found.

### Limitations

There are some limitations to consider in this study. Firstly, there may be an underestimation or misclassification of suicide data. The data used for this study was obtained from government organizations in Iran and we must acknowledge that there may be an underestimation to the true numbers in suicide within Iran, especially in the more remote provinces [18]. This is due to factors such as the social stigma towards suicide, religious sanctions and legal issues that may cause the under reporting of attempted and completed suicides by family, police, doctors or coroners [60, 61]. These factors are known to arise more commonly in provinces with lower social status.

Secondly, this study has not included some important factors that may have an impact on suicide rates within Iran due to availability of data. Mental health prevention programme implementation and access may vary greatly between provinces and are often reliable predictors of suicide rates in spatial and temporal perspectives [23]. Furthermore, the prevalence of drug abuse, psychiatric disorders, such as depression, are strong risk factors for suicide [62–65].

Thirdly, suicide mortality rates used in this analysis are crude rates and not age-standardized rates. This is because lack of access to data on age distribution of suicide mortality at the province level. However, the mortality rates are naively adjusted for age by including proportion of population age 15–24 and 65+ in the analyses.

Lastly, this analysis is an ecological study, using aggregated data at the province level, implying that the results may vary greatly within provinces themselves. Therefore, the results are subject to “ecological fallacy” and may not necessarily be applicable to smaller geographical units or at individual level. Break downs with provincial studies that assess socio-demographic characteristics in areas with similar access to mental health services may be of value in a country like Iran.

## Conclusion

This study is one of very few studies that have conducted a two-dimensional analysis of suicide with spatial and temporal variation concomitantly. The findings of the current study demonstrate that both geographic and temporal variation in suicide mortality rate are closely tied to a number of varying social conditions across provinces and over time. Further research is needed to identify the factors that can reduce the risk of suicide, such as mental health projects, especially in high-risk provinces of Iran. Greater clarity on both risk and protective factors will help prevention strategies reduce suicide rates most efficiently.

## Additional file

Additional file 1: **Table S1.** Summary statistics for all the variables include in the analysis. (XLSX 44 kb)

## Abbreviations

DALYs: disability adjusted life years
EMR: Eastern Mediterranean Region
IFMO: Iranian Forensic Medicine Organization
IRR: Iranian Rial
